# Immunophenotyping of Inflammatory Cells Associated with Schmallenberg Virus Infection of the Central Nervous System of Ruminants

**DOI:** 10.1371/journal.pone.0062939

**Published:** 2013-05-07

**Authors:** Vanessa Herder, Florian Hansmann, Peter Wohlsein, Martin Peters, Mariana Varela, Massimo Palmarini, Wolfgang Baumgärtner

**Affiliations:** 1 Department of Pathology, University of Veterinary Medicine, Hannover, Lower Saxony, Germany; 2 Staatliches Veterinäruntersuchungsamt, Arnsberg, North-Rhine Westphalia, Germany; 3 MRC Centre for Virus Research, Institute of Infection, Immunity and Inflammation, College of Medical, Veterinary and Life Sciences, University of Glasgow, Glasgow, United Kingdom; 4 Center for Systems Neuroscience, Hannover, Germany; University of Kansas Medical Center, United States of America

## Abstract

Schmallenberg virus (SBV) is a recently discovered Bunyavirus associated mainly with abortions, stillbirths and malformations of the skeletal and central nervous system (CNS) in newborn ruminants. In this study, a detailed immunophenotyping of the inflammatory cells of the CNS of affected animals was carried out in order to increase our understanding of SBV pathogenesis. A total of 82 SBV-polymerase chain reaction (PCR) positive neonatal ruminants (46 sheep lambs, 34 calves and 2 goat kids) were investigated for the presence of inflammation in the brain and spinal cord. The study focused on 15 out of 82 animals (18.3%) showing inflammation in the CNS. All 15 neonates displayed lymphohistiocytic meningoencephalomyelitis affecting most frequently the mesencephalon and the parietal and temporal lobes. The majority of infiltrating cells were CD3-positive T cells, followed by CD79α-positive B cells and CD68-positive microglia/macrophages. Malformations like por- and hydranencephaly, frequently found in the temporal lobe, showed associated demyelination and axonal loss. SBV antigen was detected in 37 out of 82 (45.1%) neonatal brains by immunohistochemistry. In particular, SBV antigen was found in 93.3% (14 out of 15 ruminants) and 32.8% (22 out of 67 ruminants) of animals with and without encephalitis, respectively. Highest amounts of virus-protein expression levels were found in the temporal lobe. Our findings suggest that: (i) different brain regions display differential susceptibility to SBV infection; (ii) inflammatory cells in the CNS are found only in a minority of virus infected animals; (iii) malformations occur in association with and without inflammation in the CNS; and (iv) viral antigen is strongly associated with the presence of inflammation in naturally infected animals. Further studies are required to explore the cell tropism and pathogenesis of SBV infection in ruminants.

## Introduction

Schmallenberg virus (SBV) represents the first member of the Simbu serogroup within the *Bunyaviridae* that causes malformations in newborn ruminants in Europe. It was first detected in Germany and other parts of Europe during the autumn of 2011 [1]. Dairy cows showed a drop of milk yield, diarrhea and fever followed by of abortions and malformations in the following parturition period [1]. Besides domestic ruminants, SBV infection was detected in a bison and in alpacas [2], [3].

SBV was discovered by metagenomic analysis of blood samples of cows from a farm in North Rhine-Westphalia in Germany [1]. This newly described pathogen belongs to a group of teratogenic arthropod borne viruses, including Akabane virus (AKV) [4], [5]. In particular, SBV belongs to the species *Sathuperi virus* within the orthobunyavirus genus and represents most likely an ancestor of *Shamonda virus* [6]. The virus infects primarily sheep, goats and cattle and is supposed to be transmitted by culicoides biting midges including *Culicoides (C.) obsoletus complex, C. dewulfi, C. scoticus and C. chiopterus* [4], [7], [8]. Indeed, the high percentage of *Culicoides spp*. positive for SBV might represent a major factor for the rapid spread of the infection throughout Europe [7]. At the end of 2012, SBV infection was found in numerous European countries including The Netherlands, Belgium, United Kingdom, Italy, France, Spain, Luxembourg, Denmark, Ireland, North Ireland and Switzerland [9] and antibodies to SBV were detected in animals originating from Austria, Poland, Sweden, Finland and Norway (www.fli.bund.de).

As of December 2012, SBV was detected in 2016 herds in Germany. Cattle farms were primarily affected followed by sheep herds and goat holdings (www.fli.bund.de). The seroprevalence of SBV decreased from the west of Germany to southern and eastern parts from more than 95% to 67% in ruminant herds [9]. In The Netherlands a seroprevalence of 72.5% in dairy cattle in the winter 2011 and 2012 was noticed [10]. Similarly, a very high percentage of bovines were infected in Belgium [11].

Until now, the mode of introduction of SBV into The Netherlands [12], Germany and/or Europe is still unclear, but several similarities are shared with the epidemiology of Bluetongue virus (BTV), another arthropod-borne virus [13]. BTV serotype 8 emerged in Europe in 2006 and was associated with congenital malformations in ruminants similar to the ones observed in SBV-infected animals [14]. A possible scenario includes the incidental transport of SBV-infected insects into Europe along imported flowers from Africa [15], [16]. However, it still remains unclear whether SBV represents truly a newly emerging virus or a virus that has not been detected before and has been brought to a new environment and a highly susceptible naïve ruminant population.

So far little is known about the mechanisms of SBV pathogenesis in fetal and adult animals. Recently, murine models were established to study determinants of SBV virulence and viral pathogenesis in detail [17]. Intracerebral inoculation of SBV in newborn NIH-Swiss mice was lethal, while a SBV mutant lacking one of its non-structural proteins (NSs) showed an attenuated phenotype [17]. Importantly, SBV-infected mice displayed neuronal infection and lesions within the CNS, characterized by cerebral malacia and vacuolation, resembling natural infection in ruminants [17]. Current assumptions about SBV pathogenesis in ruminants are frequently based on findings described for AKV and other members of the Simbu serogroup. Similarly to SBV, AKV infection causes in aborted or stillborn neonatal ruminants the so called arthrogryposis and hydranencephaly syndrome (AHS) [18].

Despite the substantial scientific achievements within the last 12 months, there is still a lack of data on SBV virulence and pathogenesis in its natural hosts. In this study we dissected the natural SBV infection in neonatal ruminants, aiming to: (i) determine the extent and distribution pattern of the inflammatory response in the CNS; (ii) evaluate the phenotype of inflammatory changes; and (iii) correlate the presence of SBV protein expression with lesion development in SBV naturally infected neonatal ruminants.

## Materials and Methods

### Animals

Eighty two formalin-fixed CNS tissues, collected between December 2011 and April 2012 derived from neonatal animals (46 sheep lambs, 34 calves and 2 goat kids) with natural SBV infection originating from North Rhine-Westphalia in Germany, were examined using hematoxylin and eosin (HE)-staining for the presence of inflammatory changes in the CNS and meninges. All neonates were positive for SBV genomic and mRNA in the CNS, meconium or blood using a reverse transcriptase quantitative polymerase chain reaction (RT-qPCR) test developed at the Friedrich-Loeffler-Institute [1]. A summary of the lesions observed in the animals used in this study are shown in Table 1. All the specimens derived from aborted, stillborn, euthanized animals or ruminants that died during the perinatal period. In addition, CNS malformations of 67 neonates (33 sheep, 33 calves, 1 goat) without encephalomyelitis were shown. This study was conducted in accordance with the German Animal Welfare Act. All animals were dead at the time of submission for necropsy in order to investigate the causes of abortion, stillbirth or perinatal death. The authors confirm that no animals were sacrificed for the purpose of this study. All animal tissues originated from the ‘Staatliches Veterinäruntersuchungsamt Arnsberg’. Some of the tissues were used in previous publications [17], [19], [20].

**Table 1:** Animals used in the study.

Animal no.	Species	Gross findings	Tissues used for PCR*
1	Goat	Arthrogryposis, deformation of vertebral column	Cerebrum, medulla oblongata, cerebellum, spinal cord
2	Calf	Hydranencephaly	Cerebrum
3	sheep	Hydranencephaly	Cerebrum
4	Sheep	Torticollis, cerebellar hypoplasia, porencephaly	Central nervous system
5	Sheep	Torticollis, porencephaly, hydrocephalus internus, cerebellar hypoplasia	Cerebrum, medulla oblongata, cerebellum
6	Sheep	Brachygnatia inferior, torticollis, arthrogryposis, porencephaly, cerebellar hypoplasia	Cerebrum
7	Sheep	Arthrogryposis, torticollis, brachygnathia inferior, hydranencephaly, cerebellar hypoplasia,	Medulla oblongata, pooled tissues (cerebellum, cerebrum, spleen)
8	Sheep	Arthrogryposis, torticollis, cerebellar hypoplasia	Medulla oblongata, pooled tissues (cerebellum, cerebrum)
9	Sheep	Arthrogryposis, torticollis, porencephaly, hydrocephalus internus, cerebellar hypoplasia	Blood
10	Sheep	Arthrogryposis, brachygnathia inferior, hydranencephaly, cerebellar hypoplasia	Blood, medulla oblongata, cerebrum
11	Sheep	Arthrogryposis, porencephaly, cerebellar hypoplasia	Blood, pooled tissues (medulla oblongata, cerebellum, cerebrum)
12	Sheep	None	Blood, pooled tissues (medulla oblongata, cerebellum, cerebrum)
13	Sheep	Arthrogryposis, torticollis, porencephaly, cerebellar hypoplasia	Blood, pooled tissues (medulla oblongata, cerebellum, cerebrum)
14	Sheep	Arthrogryposis, torticollis, porencephaly, cerebellar hypoplasia, hydrocephalus internus	Blood, medulla oblongata, cerebellum
15	Sheep	Arthrogryposis, brachygnathia inferior, cerebellar hypoplasia, porencephaly,	pooled tissues (cerebrum, cerebellum, medulla oblongata)

* all animals were SBV RT-qPCR positive.

### Histology and Histochemistry

For histology, brain and spinal cord tissue was routinely processed in paraffin wax, cut at 3 µm thickness, and stained with hematoxylin and eosin (HE). The following brain regions were evaluated using HE-stained sections: frontal, parietal, temporal and occipital lobe, hippocampus, brain stem, medulla oblongata, cerebellum, mesencephalon and the spinal cord (only from one sheep). A semi-quantitative scoring system was used to evaluate the degree of the inflammation using HE-stained sections. Meningeal infiltrates, perivascular cuffing and parenchymal cells were scored as follows: 0 cells = −; 1–3 cell layers = + (mild); 4–7 cell layers = ++ (moderate); ≥8 layers = +++ (severe) for meninges and perivascular cells and 0 cells = −; 1–19 cells = + (mild); 20–29 cells = ++ (moderate) and ≥30 cells = +++ (severe) for parenchymal cellular infiltrates per high power field (HPF) and region, respectively. The highest score of each of these was considered representative for the respective region.

The Prussian blue, Bielschowsky’s silver and von Kossa stain were performed as described previously [20], [21]. Selected CNS areas were semi-quantitatively evaluated using von Kossa-staining for the presence of parenchymal mineralization as well as Prussian blue-positive material to detect hemosiderosis. Bielschowsky’s silver impregnation was used to determine axonal density in the white matter. No reduction of axons was scored with −, whereas decreased staining of up to 1/3 =  +, 1/3–2/3 = ++ and ≥2/3 = +++ within regions of interest were diagnosed as mild, moderate and severe axonal loss, respectively.

### Immunohistochemistry

In order to characterize cellular infiltrations, T cells, B cells and microglia/macrophages were semi-quantitatively determined by investigating CD3-, CD79α- and CD68-expression, respectively. Antibodies and conditions used for immunohistochemistry are summarized in Table 2. For visualization of the antigen-antibody reaction the ABC-method (VECTASTAIN® Elite ABC Kit, Vector Laboratories) with 3, 3′-diaminobezidine-tetrahydrochloride (DAB) as chromogen was applied [22]. The following brain regions displaying the most prominent inflammatory changes as determined by HE-staining were investigated: mesencephalon, hippocampus as well as the temporal and parietal lobe. Up to 2 layers of CD3-positive, perivascular layers and up to 10 CD3-positive cells per high power field in the parenchyma were scored with + (mild), the score ++ (moderate) was given for 3–5 layers and up to 20 cells, and a score of +++ (severe) was used for ≥5 cell layers and ≥20 parenchymal cells. For CD79α-positive B cells and CD68-positive microglia/macrophages, score + (mild) was given for 1 perivascular layer and up to 10 positive cells in a 20-fold magnification. Score ++ (moderate) was used for up to 2 perivascular layers and up to 20 cells, and score +++ (severe) was used for ≥3 layers and ≥20 parenchymal cells.

**Table 2:** Antibodies and conditions applied for immunohistochemistry in formalin-fixed and paraffin-embedded brain tissue.

Antigen	Blockingserum	Pre-treatment	Dilution	Secondary antibody	Company; product number/provider
CD3	Goat	Microwave, 20 min., citrate buffer	1∶1000	Goat anti rabbit	Dako/Agilent Technologies; A0452
CD79α	Goat	Microwave, 20 min., citrate buffer	1∶60	Goat anti mouse	Dako/Agilent Technologies; M7051
CD68	Goat	Proteinase K, 10 min.	1∶20	Goat anti mouse	Dako/Agilent Technologies; M0718
MBP	Goat	–	1∶500	Goat anti rabbit	Merck/Millipore; AB980
PLP	Goat	Microwave, 20 min., citrate buffer	1∶500	Goat anti mouse	AbD Serotec; MCA839G
CNPase	Goat	Microwave, 20 min., citrate buffer	1∶100	Goat anti mouse	Merck/Millipore; MAB326
GFAP	Goat	–	1∶1000	Goat anti rabbit	Dako/Agilent Technologies; Z0334
SBV-nucleoprotein	Goat	–	1∶3000	Goat anti rabbit	Massimo Palmarini, Glasgow, UK [17]

− = no pre-treatment; MBP = myelin basic protein; PLP = proteolipid protein; GFAP = glial fibrillary acidic protein; SBV = Schmallenberg virus.

The presence of SBV was determined in all animals (n = 82), including 15 neonates with encephalitis and 67 without inflammation in the brain using a rabbit polyclonal antibody against the viral nucleoprotein as already described [17]. The brain regions with the most prominent inflammation such as hippocampus, mesencephalon as well as the parietal and temporal lobe were evaluated applying a semi-quantitative scoring system: no positive cells = −, 1–10 positive cells = + (mild), 11–20 positive cells = ++ (moderate) or ≥20 cells = +++ (severe) at 20-fold magnification in one field. The highest value was taken as representative final degree of severity of virus expression for the region of interest and related to pathologic findings including inflammation and tissue loss (e.g. porencephaly) in the respective area.

To evaluate oligodendroglial cells, marker expression in the white matter, myelin basic protein (MBP), proteolipid protein (PLP) and 2′, 3′-cyclic nucleotide 3′-phosphodiesterase (CNPase) were used and the following evaluation system was applied: no demyelination = − (100% of the myelin fibers stained positive). A loss of myelin of up to 1/3, 1/3–2/3, and ≥2/3 of the region investigated were scored as + (mild), ++ (moderate), and +++ (severe), respectively. The degree of astrogliosis was determined by using glial fibrillary acidic protein (GFAP) immunohistochemistry applying a simplified scoring system (0 =  no astrogliosis; 1 =  astrogliosis).

## Results

### Histopathology of the Brain and Spinal Cord of SBV Infected Animals

**Figure 1:**
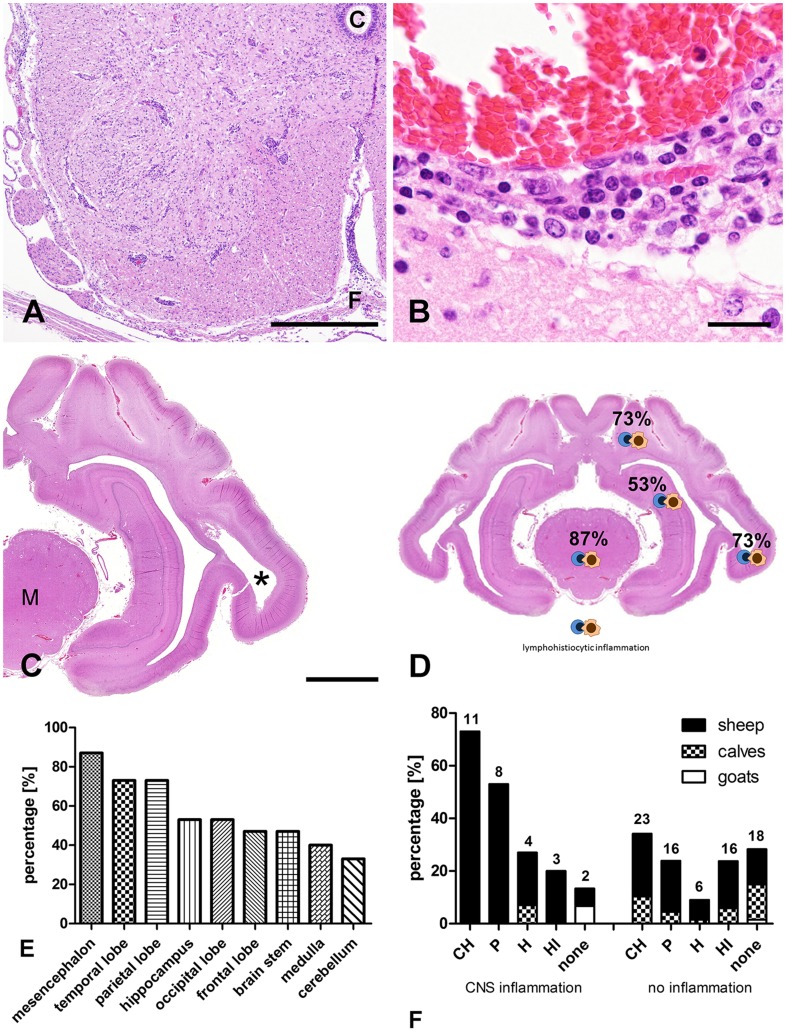
Histopathology and distribution pattern of inflammation in naturally with SBV-infected neonatal ruminants (n = 15) of the central nervous system (HE-staining). **A)** Spinal cord of a sheep displaying mild, multifocal lymphohistiocytic infiltrates in meninges, perivascular spaces and the parenchyma of gray and white matter (animal no 4; C = central canal; F = fissura mediana ventralis; bar, 1000 µm). **B)** Moderate perivascular infiltrates characterized by lymphocytes and macrophages in the brain of a SBV-infected sheep (animal no 10; bar, 20 µm). **C)** Coronal section of a brain showing the mesencephalon, hippocampus, lateral ventricle as well as parietal and temporal lobes. The temporal lobe of this sheep lamb displays a porencephaly with complete loss of the white matter in the center (asterisk; animal no 5; M = mesencephalon; bar, 5000 µm). **D)** Topographic map of a coronal brain section displaying parietal, temporal lobes, mesencephalon and hippocampus summarizing the percentages of histologic findings in all investigated animals with respected to specific brain regions. **E)** Percentages of brain regions displaying inflammatory changes in ruminants with SBV infection. **F)** Occurrence of CNS malformations in SBV-infected animals associated with (n = 15) and without inflammation (n = 72). Percentages of animals displaying cerebellar hypoplasia (CH), porencephaly (P), hydranencephaly (H), hydrocephalus internus (HI) and no malformations (none) are given. Absolute numbers of affected animals are given on top of the bar. Note, that one animal exhibited more than one malformation.

Inflammatory changes of brain and/or meninges were detected in 15 (18.3%) of 82 ruminant neonates naturally infected with SBV. More specifically, inflammation was detected in 13 out of 46 sheep lambs (28.3%), 1 out of 34 calf (2.9%) and in 1 out of 2 goat kids (50%). In order to characterize the topography of the inflammatory process, 9 CNS areas were evaluated and scored individually. Inflammatory changes were characterized by lymphohistiocytic perivascular cuffs with few plasma cells and variable numbers of cell layers and parenchymal infiltrates in the gray and white matter of the brain, spinal cord and the meninges (Fig. 1A–B). The mesencephalon was affected in 13 of 15 cases, and parietal and temporal lobes in 11 of 15 cases. The hippocampus and occipital lobes displayed inflammation in 8 out of 15 brains, whereas only 7 out of 15 animals exhibited inflammation in the frontal lobe and brain stem. The cerebellum and medulla oblongata were less often affected (5 and 6 of 15 cases, respectively) than other areas of the brain. In addition, lymphohistiocytic infiltrates were also found adjacent and distant to malformations like porencephaly (Fig. 1C). A schematic overview of the region-specific occurrence of the inflammation is given in Figure 1D–E.

Malformations like por- and hydranencephaly were detected macroscopically and histologically in animals with inflammation. Hydranencephaly was found in 4 of 15 animals with inflammation (26.6%; 3 sheep lambs, 1 calf) while 8 neonates showed porencephaly (53.3%; 8 sheep lambs) in the temporal and parietal lobes. Animals displaying hydranencephaly showed remnants of cortical tissue adherent to the meninges and a recognizable mesencephalon. The size of pores varied between 1 mm up to approximately 1 cm in diameter (in animal no. 4–6, 9, 11, 13–15). All porencephalies occurred bilateral-symmetrically and were primarily located in the temporal lobe (animal no. 5, 6, 13–15; table 1). Animal no. 4, 9 and 11 displayed porencephaly in both, the temporal and parietal lobes (multicystic encephalopathy). Porencephaly was associated with adjacent accumulations of Gitter cells (macrophages/microglia with a foamy cytoplasm indicative of phagocytic activity). Furthermore, loss of tissue resulting in porencephaly was associated with remnants of CNS parenchyma consisting of tissue strands partly connected to the periphery of the pores within the lumen. 11 (73.3%) sheep lambs had cerebellar hypoplasia, 3 (20%) sheep lambs showed a hydrocephalus internus and 2 animals (animal no. 1 and 12; 13.3%) lacked CNS malformations. Animals without inflammatory changes in the CNS (n = 67) displayed a great variety of malformations, like porencephaly, hydranencephaly, hydrocephalus internus, cerebellar hypoplasia. A comparison of the occurrence of malformations in animals with and without CNS inflammation is shown in Figure 1F.

### Phenotyping of Inflammatory Cells

**Figure 2:**
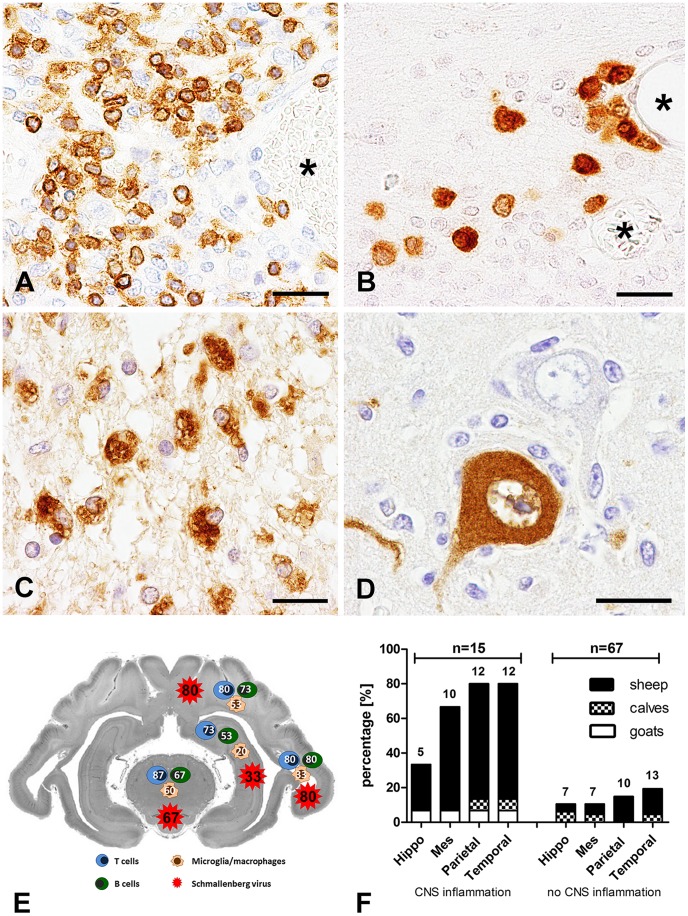
Immunophenotyping of immune cells, detection of virus protein and distribution of immune cells and virus protein in the brain of SBV-infected ruminants (n = 15). **A)** Detection of CD3-positive T cells in the perivascular space and adjacent tissue of a goat kid (animal no 1; asterisks = vessel lumen; bar, 20 µm). **B)** CD79α-positive B cells are located diffusely in the brain parenchyma and around vessels (asterisks) in a sheep lamb (animal no 11; bar, 20µm). **C)** SBV-infection caused in some animals pore-associated Gitter cell infiltration. These cells showed prominent CD68-expression (sheep lamb no 4; bar, 20 µm). **D)** SBV protein was diffusely distributed in the cytoplasm and processes of neurons (goat kid, animal no 1; bar, 20 µm). **E)** Distribution and percentage of affected brain regions using immunophenotyping of inflammatory cells with special emphasis upon T cells (CD3), B cells (CD79α) and microglia/macrophages (CD68) as well as SBV antigen distribution. Individual numbers within depicted cell types indicate the percentages of animals displaying the specific cell types in the respective brain regions. **A–E)** Visualization: ABC-method (VECTASTAIN® Elite ABC Kit, Vector Laboratories) with 3, 3′-diaminobezidine-tetrahydrochloride (DAB) as chromogen. **F)** Occurrence of SBV-antigen in the CNS of 82 naturally infected neonatal ruminants with (n = 15, 14 positive animals, 93.3%) and without inflammation (n = 67, 22 positive animmals, 32.8%). Percentages of animals showing virus protein in the hippocampus (Hippo), mesencephalon (Mes), temporal lobe (Temporal) and parietal lobe (Parietal) are displayed. Absolute numbers of affected animals are given on top of the bar. Note, that one animal may exhibit more than one SBV antigen positive region in the CNS.

Immunophenotyping of cellular infiltrates in the brain parenchyma and meninges revealed that the vast majority of infiltrating cells were CD3-positive T cells. Inflammation was detected by CD3-immunohistochemistry especially in the mesencephalon. 13 and 12 animals showed parenchymal T cell infiltrates in the mesencephalon and parietal and temporal lobes, respectively. Only 11 brains showed T cells in the hippocampus. Interestingly, mild to moderate perivascular cuffing of CD3^+^ cells was detected in 9 cases only within the mesencephalon (Fig. 2A). Parietal and temporal lobes showed perivascular cuffing of T cells in 11 ruminants, whereas perivascular T cell cuffs in the hippocampus were only detected in 6 animals.

CD79α-positive B cells were found to a lesser extent in the brains of affected animals. Slight to moderate amounts of B cells were located perivascularily and within the parenchyma (Fig. 2B). Only animal no. 4 showed severe B cell infiltrations. In 12 animals, B cells were found in the temporal lobe representing the brain region with the highest frequency of CD79α^+^ cells. The parietal lobe and mesencephalon were affected in 11 and 10 cases, respectively. The hippocampus showed B cell infiltrations in only 8 cases. B cell infiltration was detected in association with and without malformations.

A mild amount of microglia/macrophages expressing CD68 was found within the mesencephalon in 9 out of 15 investigated animals. Areas displaying malformations, including the temporal and parietal lobe showed positive microglia/macrophages in 5 and 8 cases, respectively. CD68-positive cells were only found in the hippocampus of 3 animals. In areas of malformations the cytoplasm of Gitter cells was strongly positive for CD68 (porencephaly, Fig. 2C).

### Distribution of Schmallenberg Virus

The vast majority of SBV infected cells showed neuronal morphology (Fig. 2D) with long processes resembling axons and dendrites. Viral antigen was present in 37 out of 82 (45.1%) neonatal brains. 14 of 15 animals (93.3%) with encephalitis displayed SBV nucleoprotein in mesencephalon, temporal and parietal lobes as well as the hippocampus. Virus protein was not observed in 1 lamb (animal no. 6) in any of the brain regions investigated despite detectable inflammation. In contrast, only 32.8% (22 out of 67) of brains in animals without inflammation were positive for SBV antigen.

In general, SBV antigen was found simultaneously in various brain regions. The temporal and parietal lobes in the inflamed brains showed in 80% of the cases the highest numbers of SBV infected cells. In addition, SBV antigen-expression was strongly associated with malformations like porencephaly. The mesencephalon, the most frequently inflamed brain region, displayed virus-positive cells in 10 out of 15 cases (66.7%), while the hippocampus was affected in 5 cases (33.3%). In brains without inflammation, SBV protein was found in 13 (19.4%) and 10 (14.9%) cases in the temporal and parietal lobes, respectively. In 7 animals, the hippocampus or the mesencephalon were positive for SBV-protein in non-inflamed brains only. A schematic overview about the region-specific occurrence of inflammation and SBV protein detected by immunohistochemistry is given in Figure 2E. In addition, percentages of virus protein in the hippocampus, mesencephalon, parietal and temporal lobes of animals with and without inflammation is comparatively displayed in figure 2F.

### Axonal Damage, Myelin Loss and Astrogliosis

**Figure 3:**
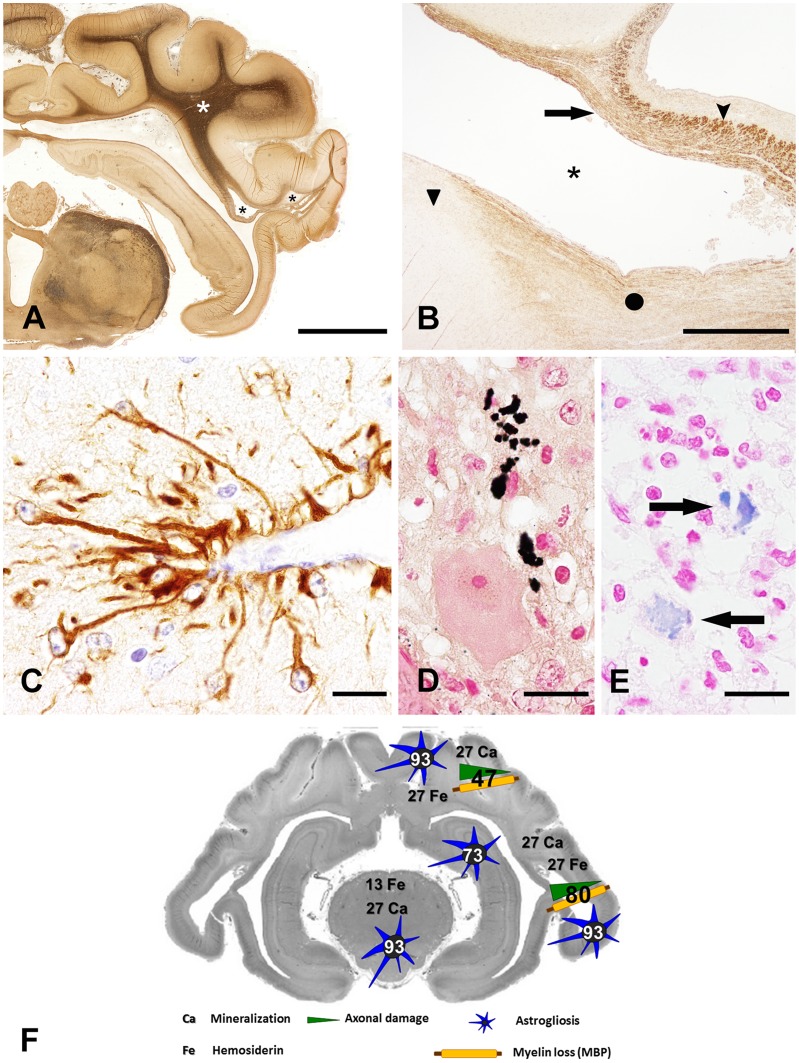
Animals naturally infected with SBV (n = 15) show pore-associated axonal damage and demyelination. Astrogliosis and deposition of hemosiderin and calcium is also shown. **A)** Bielschowsky’s silver impregnation indicating axonal loss within a pore in the temporal lobe (black asterisks). The remaining white matter surrounding the pore shows a light brown staining of Bielschowsky’s silver impregnation indicating further axonal loss. In contrast, the non-affected white matter with normal axonal density is characterized by a dark brown staining (white asterisk) within the same tissue section (animal no 14; bar, 5000 µm). **B)** Pore associated demyelination (black asterisk) as shown by myelin basic protein immunohistochemistry in the temporal lobe of a neonatal sheep. The degree of severity of myelin loss varied from mild (arrow), moderate (closed circle) to severe (triangular). Note region of intact myelin basic protein expression consists of a dark brown immunoreaction (arrow head; animal no 5, bar, 1000 µm). **C)** Prominent immunoreactive processes of astrocytes are found especially in association with the vessel walls (GFAP staining, animal no 14; bar, 20 µm). **D)** Deposition of von Kossa positive, extracellular, coarsely granular material (mineralization), presumably due to tissue loss and necrosis (sheep lamb, animal no 12; bar, 20 µm). **E)** Deposition of Prussian blue, extracellular, coarsely granular material (iron) next to a pore, interpreted as hemosiderin is presumably a sequel of old hemorrhages (arrows; animal no 4; bar, 20 µm). **F)** Summarized presentation of axonal damage, demyelination, astrogliosis, mineralization and hemosiderosis. Axonal and myelin loss were present only in close proximity to por- or hydranencephaly, whereas mineralization, hemosiderosis and astrogliosis were found in brain regions exhibiting malformations or inflammation. Individual numbers within depicted cell types indicate the percentages of affected animals in the respective region of the brain.

For the evaluation of axonal density and damage, the Bielschowsky’s silver impregnation was applied and changes were semi-quantitatively scored in the white matter. Axonal density was moderately to severely reduced adjacent to areas of tissue destruction such as porencephaly (Fig. 3A), whereas no obvious axonal changes were detected in areas with inflammation such as the mesencephalon, indicating axonopathy secondary to tissue destruction and not directly related to virus infection and inflammation.

In cases of malformations with por- and hydranencephaly, a slight to severe reduced staining intensity of myelin proteins such as MBP (Fig. 3B), PLP, and CNPase was detected in the surrounding white matter. No demyelination was found in the mesencephalon and hippocampus. Both regions lacked malformations and were characterized by inflammatory changes only.

The mesencephalon as well as temporal and parietal lobes of 14 animals (all animals except animal no. 8) showed increased GFAP-expression. The hippocampus was affected by astrogliosis only in 11 cases. The positively stained cells were diffusely distributed and 7 animals had an increased amount of GFAP-positive cells around blood vessels (Fig. 3C).

### Mineralization and Hemosiderosis

Mineralization of CNS parenchyma as detected by von Kossa staining was found in 6 animals (animal no. 2–4, 7, 12, 14, 15). The temporal and parietal lobes as well as the mesencephalon were affected to the same extent. The deposition of von Kossa positive material was present at the rim of the pores (Fig. 3D) and also within regions of inflammation, e.g. in the mesencephalon.

Iron deposits detected by Prussian blue were found in 5 animals (animal no. 3, 4, 7, 8, 12; Fig. 3E). The parietal and temporal lobes were most frequently affected (4 cases each), while positive material in the mesencephalon was only found in 2 animals. Interestingly, deposition of calcium and iron was found frequently in regions displaying large areas of tissue destruction, like por- and especially hydranencephaly. A schematic overview on the results regarding axonal damage, demyelination, astrogliosis, hemosiderosis and mineralization is given in Figure 3F.

## Discussion

SBV is a teratogenic virus, similarly to other viruses, including AKV, BTV and pestiviruses like bovine virus diarrhea virus (BVDV), border disease virus (BDV) and classical swine fever virus (CSFV) [14], [18], [23]. All these virus infections show similar gross findings including cerebellar hypoplasia, por- or hydranencephaly and skeletal malformations like brachygnathia and arthrogryposis of *in utero-*infected neonates [20], [23], [24]. Furthermore, histopathology frequently reveals non-suppurative meningoencephalomyelitis with necrosis of the brain [20], [25], [26], [27], [28], [29].

However, so far little was known about pathogenesis and associated pathology of natural SBV infection. Therefore, the aim of the present study was to determine the prevalence and topography of CNS inflammation, occurrence of astrogliosis, demyelination, axonal damage as well as mineralization and hemosiderosis and their relation to virus protein distribution in the CNS of naturally SBV-infected ruminants.

The present study focused on 15 out of 82 animals that displayed lymphohistiocytic meningoencephalomyelitis. Interestingly, inflammation occurred more frequently in small ruminants (13 lambs out of 46 animals = 28.3%, 1 out of 2 goats = 50%) compared to calves (1 out of 34 animals = 2.9%). Grossly, CNS malformations of SBV-infected ruminants included hydranencephaly, porencephaly, cerebellar hypoplasia and internal hydrocephalus as described before [20]. The same variety of malformations occurred in the inflamed and non-inflamed CNS of SBV-infected neonates. Furthermore, these malformations occurred in similar high percentages in animals with and without CNS inflammation. Cerebellar hypoplasia and porencephaly represented the most frequently detected malformations in both groups. Malformations in the CNS of SBV-infected animals occurred in combination with and without inflammation indicating the contribution of a specific factor to the development of inflammation. The anatomy of the placenta in ruminants inhibits the transport of immune cells and/or antibodies from the pregnant animal to the fetus [30]. Therefore, any fetal immune response depends upon the state of development and maturation of the fetal immune system. Furthermore, inflammation in SBV-infected animals as in any other fetal ruminant disease depends on the time of infection. The development of the fetal bovine immune system, characterized by the cellular population of the thymus and lymph nodes and the production of antibodies, starts at 41 days post gestation and continues approximately until day 175 of gravidity [30]. In lambs, it stars at 19 days post gestation and lasts until 115 days after conception [30]. During this time period, viral antigen recognition by the fetal immune system is possible and may result in an inflammatory response such as encephalitis. Infection of the fetus before this time period could result in immune tolerance, as described for bovine virus diarrhea virus [30] and the absence of inflammation. In addition to the gross lesions, porencephaly was also detected by light microscopy mainly in the temporal lobe. In severe cases, the parietal lobe was also affected by formation of such cavities. In humans, the occurrence of multiple cysts in the brain due to a hypoxic-ischemic pathogenesis has been described. This entity is termed *multicystic encephalopathy* [31], [32]. The pathological changes associated with SBV infection in ruminants seem to fit the description of multicystic encephalopathy. Furthermore, the virus-induced vascular lesions observed in SBV as well as to AKV and BTV infection could explain the development of por- and hydranencephaly [33], [34]. In addition, hemosiderosis, mineralization and vasculitis in the CNS have been detected in BTV infections [34]. The blood vessel damage, associated CNS hemorrhages, hemosiderosis and mineralization observed in SBV-infected animals is supposed to be the cause of por- and hydranencephaly. This in turn would fit the description of porencephaly type I, which is caused by vascular disruption leading to CNS damage with necrosis, hemorrhages and at late stages to por- and hydranencephaly or multicystic encephalopathies [34], [35]. This is in contrast to porencephaly type II, which represents a developmental disease characterized by the inability of damaged neuronal cells to migrate within the cortex [35].

Beside porencephaly, a lymphohistiocytic meningoencephalomyelitis characterized by perivascular cuffing and parenchymal T cells, B cells and microglia/macrophages was observed in SBV-infected neonates. CD3-positive T cells represented the dominating inflammatory cell type, while CD79α–positive B cells and CD68-positive microglia/macrophages were less often detected.

In general, inflammation was most frequently detected within the mesencephalon of SBV-infected animals while the temporal and parietal lobes most frequently exhibited bilateral-symmetrical malformations like porencephaly. Similar CNS lesions, like hydranencephaly and porencephaly in the cerebrum, were described for transplacentaly infected BVDV calves, and fetuses with an *in utero*-infection of Cache valley virus [36], [37]. Non-suppurative encephalomyelitis is characteristic of fetuses infected with AKV [38]. In the present study, mineralization and hemosiderosis (iron deposition) were frequently found in the parietal and temporal lobes, areas displaying frequently porencephaly. Dystrophic mineralization in areas with tissue loss due to SBV-infection represents a finding most likely due to virus-induced tissue destruction, which has also been described in AKV-infected fetuses [39], [40], [41], [42]. Hemosiderosis, possibly due to hemorrhages has also been described for porencephalies due to virus infections [43]. Both, mineralization and hemosiderosis were more frequently present in cases with por- and hydranencephaly. The relative low numbers of animals with mineralization (6 animals) and with hemosiderosis (3 animals) and the overall low extent of calcium and iron deposition in the inflamed or malformed CNS parenchyma indicates that widespread hemorrhages and severe tissue destruction were rare events or no longer detectable. Interestingly, it was shown in a mouse model for SBV-infection, that malacia of the brain was detected in the acute phase of the infection [17]. Brain malacia could represent a primary lesion resulting later in time in dystrophic mineralization and hemosiderosis.

SBV positive cells showed neuronal morphology as previously described [17] and as also seen in AKV and BVDV [40], . Importantly, the same cell tropism of SBV was also observed in newborn NIH-Swiss mice intracerebrally infected with SBV [17]. The detection of SBV by immunohistochemistry allowed a correlation between the presence of viral antigen and the occurrence of lesions. The highest number of SBV-positive cells were detected in the cerebral cortex, in particular within the temporal and parietal lobes as also described for BVDV infection of calves [44]. However, the hippocampus was rarely affected in SBV-infected ruminants in contrast to BVDV-infections [44]. Similar to Cache valley virus infection in ovine fetuses, SBV antigen is primarily detected in areas with tissue damage like necrosis and inflammation [45]. Interestingly, investigations on experimental Cache valley virus infection revealed that intense occurrence of virus antigen decreased over time to scattered areas containing few positive cells only [45]. Although the time point of infection is unknown in the present study, data suggest that virus antigen expression and inflammation may occur at the same time and both seemed to decrease with progression of the disease as described for Cache valley virus infection [45]. Similarly, 93.3% (14 out of 15) of animals with inflammation showed virus protein in the CNS and only 32.8% (22 out of 67) of ruminants without encephalitis revealed SBV antigen in the CNS. Though the process of viral clearance has not been investigated in the present study, the observed findings suggest several mechanisms. Whether B cells and plasma cells contribute to viral clearance remains a possibility and needs to be further investigated. Similarly, B cells and plasma cells as markers for chronic inflammation were associated with viral clearance in various CNS diseases including distemper [46]. In the present study, the fact, that nearly all animals with encephalitis displayed virus antigen in the brain and animals lacking CNS inflammation were virus positive in approximately 35% of the cases only, indicates a close interaction between virus expression and initiated immune response. However, viral persistence without associated immune response seems to be another mode of action in some animals. This indicates, that fetal SBV infection seems to represent a highly complex pathogenic process influenced by the age of the fetus at the time of infection, maturation of immune system and other factors such as virus strain, environment and host genetic background. Similarly, persistent infection has been described for bovine virus diarrhea virus [47].

Demyelination due to SBV infection was severe in areas displaying malformations (por- and hydranencephaly) while it was not detected in areas displaying only inflammation. Therefore, myelin loss can be directly linked to virus-induced tissue damage. In SBV-infected animals axonal damage was primarily found in the temporal lobes with porencephaly. Thus, demyelination in SBV-induced lesions in the CNS is interpreted as a secondary event due to tissue destruction and axonal damage, representing the so-called ‘by-stander demyelination’ [48]. There was no indication of virus-induced primary axonal injury that may lead to demyelination as described in the “Inside-Out model” [49]. Other CNS regions displaying only inflammation such as the mesencephalon lacked axonal pathology as described for other viral diseases such as canine distemper [50].

The time point of infection of the pregnant dam or cow is an important feature in the development of fetal inflammation and malformations. However, the exact time point of infection of the present cases remains unknown. Interestingly, only 1 calf showed inflammation in the CNS compared to 13 lambs. This discrepancy between the species is a puzzling observation and could be due to the gap between infection and parturition between sheep lambs and calves. It is assumed, that the teratogenic SBV infection might take place between the 4^th^ and 6^th^ week of gestation in sheep and before the 16^th^ week in cows [3]. Consequently, the time between infection and parturition varies from 3 to 5 months between sheep and bovines, respectively. It is possible that by the time of birth and/or abortion lambs still show the late phase of the residual inflammatory lesions whereas changes have been resolved in bovines. However, this assumption needs to be experimentally addressed. Interestingly, it has been shown for AKV that infection of pregnant goats compared to cows resulted in a higher percentages of encephalitis [38].

In summary, different etiological agents for the differential diagnosis of skeletal and CNS malformations in neonatal ruminants have to be considered depending on the geographical area and the epidemiological situation. Since 2011, SBV represents an important causative agent of arthrogryposis and hydranencephaly in Europe. The present study revealed that the temporal and parietal lobes were most frequently affected (porencephaly) and this correlated with the presence of the highest levels of viral proteins. In addition, the mesencephalon was the most frequently inflamed region of the brain in naturally SBV-infected neonatal ruminants and calcium and iron deposition as well as astrogliosis were detected in the mesencephalon and cortical lobes. Inflammation in the CNS occurred in combination with and without malformation indicating that there has to be a specific factor causing inflammation independently and additionally from por- and hydranencephaly. Furthermore, axonal loss and demyelination can be considered as secondary processes associated with malformations caused by SBV. Taken together, SBV infection of the CNS seems to cause tissue destruction and hemorrhages as determined by the detection of secondary processes such as hemosiderosis, mineralization and porencephaly, especially in the temporal and parietal lobe. Neurons are the primary place of replication of SBV and whether other cells were also affected needs to be investigated in future studies, especially in the early phase of the infection. It seems that in brain regions with no tissue destruction but with high levels of viral expression such as the mesencephalon, the virus triggers a prominent immune response. It is possible that the outcome of SBV infection in ruminants is the result of a combination of factors including the infected region of the brain, time of infection and virus species-specific interactions.
